# Visualization and analysis of medically relevant tandem repeats in nanopore sequencing of control cohorts with pathSTR

**DOI:** 10.1101/gr.279265.124

**Published:** 2024-11

**Authors:** Wouter De Coster, Ida Höijer, Inge Bruggeman, Svenn D'Hert, Malin Melin, Adam Ameur, Rosa Rademakers

**Affiliations:** 1 Applied and Translational Neurogenomics Group, VIB Center for Molecular Neurology, VIB, 2610 Antwerp, Belgium;; 2 Department of Biomedical Sciences, University of Antwerp, 2610 Antwerp, Belgium;; 3 Department of Immunology, Genetics and Pathology, SciLifeLab, Uppsala University, 751 85 Uppsala, Sweden;; 4 Neuromics Support Facility, VIB Center for Molecular Neurology, VIB, 2610 Antwerp, Belgium

## Abstract

The lack of population-scale databases hampers research and diagnostics for medically relevant tandem repeats and repeat expansions. We attempt to fill this gap using our pathSTR web tool, which leverages long-read sequencing of large cohorts to determine repeat length and sequence composition in a healthy population. The current version includes 1040 individuals of The 1000 Genomes Project cohort sequenced on the Oxford Nanopore Technologies PromethION. A comprehensive set of medically relevant tandem repeats has been genotyped using STRdust and LongTR to determine the tandem repeat length and sequence composition. PathSTR provides rich visualizations of this data set and the feature to upload one's data for comparison along the control cohort. We demonstrate the implementation of this application using data from targeted nanopore sequencing of a patient with myotonic dystrophy type 1. This resource will empower the genetics community to get a more complete overview of normal variation in tandem repeat length and sequence composition and, as such, enable a better assessment of rare tandem repeat alleles observed in patients.

Tandem repeats are head-to-tail direct repetitions of a DNA motif, which can be repeated exactly, with motif interruptions or an entirely different sequence composition for some alleles. Early methods of detection involved (repeat-primed) PCR followed by fragment-length analysis using capillary electrophoresis, Sanger sequencing, and Southern blotting. These techniques are low throughput, are labor intensive, and do not fully describe all repeat properties. Short-read sequencing technologies have difficulty in correctly determining the allele size, especially as the repeat length gets longer than the read length, but some specialized methods have enabled population-wide tandem repeat genotyping (Dolzhenko et al. 2020; Dashnow et al. 2022; Ziaei Jam et al. 2023), allowing imputation and the identification of tandem repeats relevant for traits and diseases (Manigbas et al. 2024). Increased resolution, however, is offered by long-read sequencing technologies such as nanopore and Pacific Biosciences (PacBio) sequencing, which enables direct observation of the repeat's length, sequence composition, and DNA methylation (Tanudisastro et al. 2024). The field has yet to mature fully, and a handful of tools for genotyping, benchmarking, characterization, and comparison have been developed only recently (Chiu et al. 2021; Ren et al. 2023; Dolzhenko et al. 2024; English et al. 2024; Ziaei Jam et al. 2024), without an independent assessment, identification of best practices, or development of population-scale tandem repeat databases.

In recent years, these long-read technologies have led to multiple novel discoveries of repeat loci associated with human disease (Rafehi et al. 2023; Tan et al. 2023; Cortese et al. 2024). To date, 68 repeats are associated with human diseases and summarized in STRchive (Hiatt et al. 2024), although not all of these are firmly established as pathogenic. This is the set of repeats we consider “medically relevant” in the remainder of this paper. However, we anticipate this is only the tip of the proverbial iceberg of pathogenic tandem repeats. Both the repeat length and its composition are crucial determinants of the pathogenic potential of a specific repeat allele (Rajan-Babu et al. 2024). A database of tandem repeat genotypes is beneficial to accurately assess an expanded allele's pathogenic potential versus a common population polymorphism, in which more common alleles are unlikely to be pathogenic for a patient with a rare disease. Recently, long-read sequencing technologies have matured sufficiently to apply to population-scale sequencing projects (Beyter et al. 2021; De Coster et al. 2021; Noyvert et al. 2023; Gustafson et al. 2024). The genomics community has a long-standing tradition of making data freely available, which greatly benefits the interpretation of variants identified in patients, especially in the context of rare diseases.

In this work, we describe pathSTR, a database and web app for the visualization of repeat length and sequence composition of medically relevant tandem repeats, also equipped with options to compare genotypes from other individuals (e.g., patients of interest) with the control cohort. At the time of writing, the database consists of genotypes of 1040 individuals from The 1000 Genomes Project (The 1000 Genomes Project Consortium 2015), sequenced on the Oxford Nanopore Technologies (ONT) PromethION in two initiatives (Noyvert et al. 2023; Gustafson et al. 2024; Schloissnig et al. 2024) and aligned to both the GRCh38 reference genome and the CHM13 telomere-to-telomere (T2T) assembly (Nurk et al. 2022). This resource will be extended to more individuals when additional population sequencing projects are released. The repeats are genotyped using LongTR (Ziaei Jam et al. 2024) and STRdust, two recently developed tandem repeat genotypes for long-read sequencing. We provide this data set to the genomics community for the improved interpretation of tandem repeat alleles. PathSTR is available at https://pathstr.bioinf.be.

## Results

### Genotyping medically relevant tandem repeats

LongTR (Ziaei Jam et al. 2024) and STRdust were used for genotyping the medically relevant tandem repeats from CRAM files over FTP using the curl feature of htslib (Bonfield et al. 2021). Although an in-depth benchmark is outside the scope of this work, we compared both genotypers with the HG002 tandem repeat benchmark (English et al. 2024), demonstrating excellent concordance for tandem repeat lengths for the repeats of interest (Supplemental Fig. S1). We also observe a high correlation (R = 0.8) when comparing the entire cohort's genotypes for STRdust and LongTR, with STRdust reporting longer repeats for a small set of alleles (Supplemental Fig. S2). PathSTR is flexible toward the data set to visualize, and as such, newly developed repeat genotypers that provide the repeat allele length and sequence can be integrated with pathSTR later, as well as alignments against other reference genomes. We also identified a high correlation (R = 0.95) when comparing genotypes for reads aligned to the GRCh38 reference with reads aligned to the T2T assembly (Supplemental Fig. S3). However, calculating the correlation per repeat locus shows a high variation in accuracy. Although most give identical genotypes, others, such as the *HOXA13* repeat loci, show poor accuracy, presumably owing to differences in the primary sequence between the two assemblies, differences in mappability for the locus, or inaccuracies in the repeat coordinates in the T2T assembly (Supplemental Fig. S4).

### PathSTR visualization of repeat length and sequence composition

**Figure 1.**
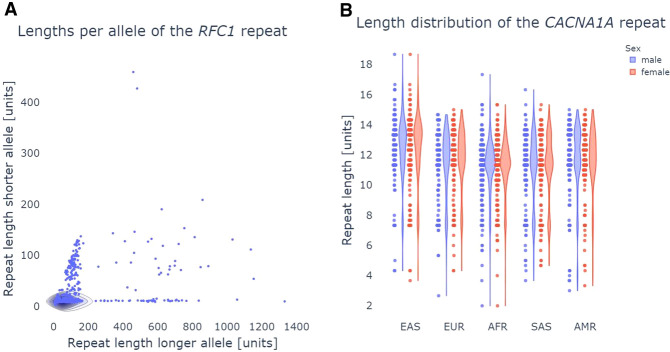
pathSTR visualization of the repeat length. (*A*) Scatter plot length visualization of the *RFC1* repeat, comparing, per individual, the longer allele against the shorter. Pathogenic repeats in *RFC1* have a recessive inheritance with pathogenicity depending on the motif composition, so observing longer alleles in the healthy population is not unexpected. (*B*) Violin and swarm plots showing the *CACNA1A* repeat length, split by The 1000 Genomes Project self-reported geographical ancestry (AMR), admixed Americans; (EUR) Europeans; (EAS), East Asians; (AFR), Africans; (SAS), South Asians, and sex, showing the pathogenic repeat length cutoff from STRchive with a red horizontal line. This locus shows longer repeat alleles are found in individuals of East Asian ancestry and shorter repeat alleles for individuals of African ancestry.

**Figure 2.**
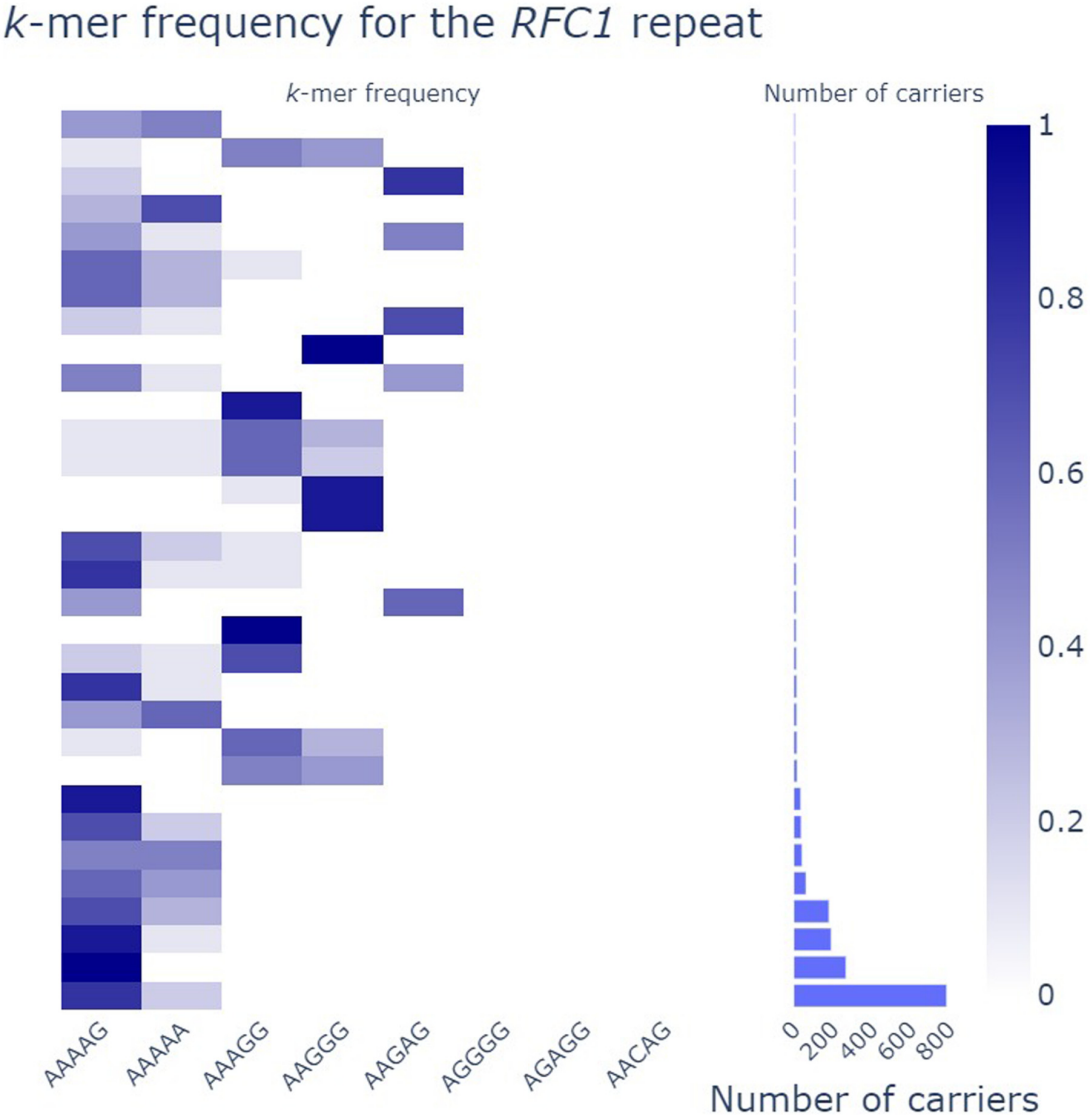
pathSTR *RFC1* composition visualization collapsed by motif, showing a marginal histogram to show the size of the groups while requiring at least five alleles per group.

**Figure 3.**
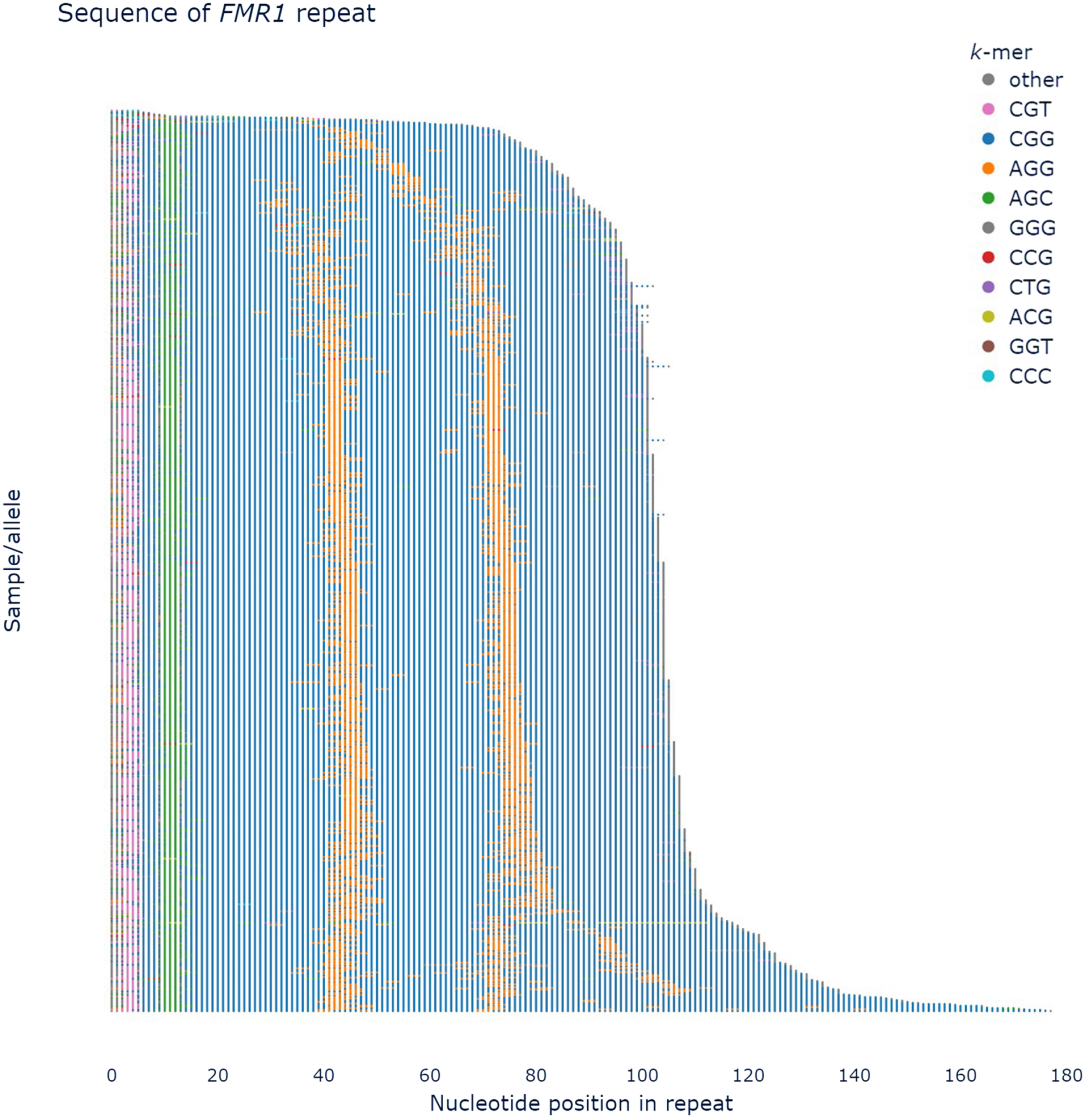
pathSTR visualization of a *sequence* plot of the *FMR1* repeat, showing colors for the most frequently seen motifs and gray for everything else, sorted by length. AGG motif interruptions (orange) between the CGG units (blue) can be observed.

The pathSTR web app (https://pathstr.bioinf.be) shows the variation in tandem repeat length (Fig. 1) and sequence composition (Figs. 2, 3) across a large cohort of control individuals. Genotyping a repeat allele required the support of two or more reads to maximize the sensitivity of observing long alleles at limited sequencing depth. Detailed information for each genotype is available for further evaluation in the web app, as well as the number of supporting reads and an IGV visualization of the long-read alignment. All plots show both alleles for an individual, except for genotypes on Chr X for individuals with one copy of the X Chromosome.

Length plots, showing either the total length of the repeat or the difference with the reference genome, can be changed to show the pathogenic length cutoff (as obtained from STRchive), perform a log-transformation of the repeat length, show a density plot, and split the individuals per The 1000 Genomes Project self-reported geographical ancestry (“superpopulation”) and/or sex. Allele lengths can be shown orthogonally (Fig. 1A) or using a strip/violin plot (Fig. 1B). The sequence composition of each repeat allele is visualized in three ways: either “raw” (showing the frequency of each repeat motif), “collapsed” (grouping samples with similar motif frequencies) (Fig. 2), or “sequence” (showing the per-allele sequence of the 10 most frequently observed motifs across the repeat length) (Fig. 3). The “sequence” mode of visualization also allows the identification of motif interruptions, such as known AGG interruptions, every nine to 10 CGGs in *FMR1* alleles associated with increased repeat stability (Fig. 3; Villate et al. 2020). The “sequence” visualization mode can also show the pathogenic length cutoff obtained from STRchive. We also provide aSTRonaut, a stand-alone companion command line script, to create “sequence”-type visualizations with additional flexibility for showing motifs of interest, optionally with motifs of different lengths. The aSTRonaut Python script can be found in the pathSTR repository at GitHub (https://github.com/wdecoster/pathSTR/blob/main/scripts/aSTRonaut.py).

### PathSTR to evaluate pathogenic repeats

**Figure 4.**
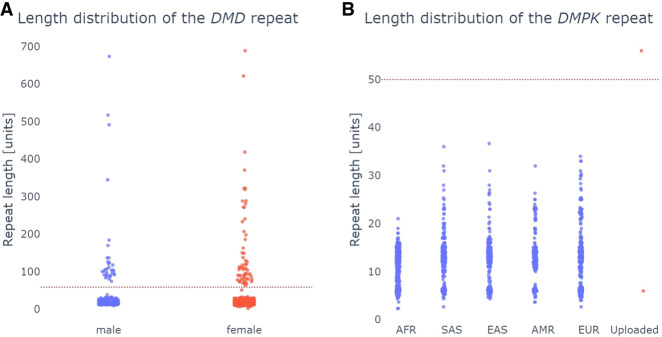
Investigating the role in disease of tandem repeat length variation in *DMD* and *DM1* (*A*) The repeat length in the *DMD* gene shows that many individuals in the general population have lengths above the proposed pathogenic length at 59 bp (red dotted line), suggesting it is not pathogenic. As *DMD* is on the X Chromosome, female samples have twice as many alleles as men. (*B*) Length visualization of the *DMPK* repeat, showing the uploaded data of the DM1 patient obtained using Cas9-enrichment, with the pathogenic cutoff indicated with a red dotted line.

STRchive includes some tandem repeat loci for which there is conflicting evidence for the association of this repeat expansion with a disease. One of these is a TTC repeat expansion in *DMD* linked to Duchenne muscular dystrophy, for which it was suggested that an allele of 59 or longer repeat units is pathogenic (Kekou et al. 2016). Investigation of this repeat using pathSTR (Fig. 4A) supports the notion that the frequency of expanded alleles is too high to be causally linked to an early-onset condition, suggesting it is not a sufficient factor to explain the disease in the family in which the expansion was described. Notably, the presence of a specific allele in a healthy control does not exclude a role in disease, as a pathogenic repeat expansion in *HTT* can be observed for HG02275 (with [CAG]_45_ and [CAG]_44_ according to, respectively, STRdust and LongTR) (Supplemental Fig. S5). A comparable expansion has already been described (Akçimen et al. 2021). Although a CAG repeat of 40 or more motifs is expected to be fully penetrant, it is possible that this participant was too young to have developed symptoms at the time of sampling (Depienne and Mandel 2021).

PathSTR enables users to upload tandem repeat genotypes generated using STRdust and LongTR from their own data sets, for example, patients of interest, and will display those next to the control cohort. For security and privacy, the uploaded data are only processed in memory and thus gets removed when the user closes the browser or refreshes the page. Figure 4B shows an example of comparing Cas9-targeted resequencing data (see Methods) (Gilpatrick et al. 2020) for a patient with myotonic dystrophy type 1 (DM1) with a *DMPK* expansion. PathSTR shows that the *DMPK* repeat length in this patient is indeed above the pathogenic line and is a length outlier compared with the rest of the cohort (*z*-score = 9.5).

## Discussion

PathSTR is the first database and visualization tool for tandem repeat genotypes from long-read sequencing. We provide the data of medically relevant tandem repeats generated with publicly available long-read sequencing data from control populations in a highly informative web application with rich, customizable, and dynamic visualizations of tandem repeat length and sequence composition. Length estimates and visualization for disease-associated loci from short-read sequencing are already available in an extremely large cohort in the gnomAD browser. This, however, does not provide the same resolution as long reads for the repeat sequence composition, and short-read methods have been shown to underestimate expanded alleles (Mohren et al. 2024). PathSTR also enables users to upload other data to visually and statistically compare against the database.

We prioritized sensitivity to detect long alleles for the pathSTR database, as the sequencing data of The 1000 Genomes Project used in this work were not uniformly sequenced to a high depth. This limited number of supporting reads (minimally, two) for some repeat alleles may lead to lower accuracy of the obtained consensus sequence owing to random sequencing noise or may lead to an estimate of the repeat length skewed owing to somatic variation. For example, Supplemental Figure S6 shows the per-read “sequence” visualization generated by the aSTRonaut.py companion tool, indicating read-to-read variation. Still, any read is a reasonable approximation of the actual allele, with greater accuracy obtained from generating the consensus sequence of two or more reads. Users should additionally be mindful of potential systematic errors from nanopore sequencing, that is, variation in longer homopolymer tracts.

We have demonstrated that STRdust and LongTR result in comparable results, which agrees with benchmarking values. However, an independent and more rigorous evaluation in this developing field is required. A first step toward this goal was already taken by compounding a tandem repeat catalog, assessing the tandem repeat variation for the HG002 Genome in a Bottle sample, and developing tools for comparison of the methods (English et al. 2024). It appears not all repeat loci are equally well genotyped in the GRCh38 reference and the T2T assembly, requiring further follow-up as more and more genome sequencing projects adopt alignment to the T2T assembly.

Although pathSTR should already greatly facilitate the interpretation of tandem repeats, we must stress that expert knowledge is still required to interpret results correctly. The pathogenic length, as obtained from STRchive, must be evaluated in light of the available data and may have to be revised when larger groups of patients and controls are sequenced. Another caution reason is that other repetitive sequences often flank tandem repeats, as is the case for *HTT*, in which both repeats are often genotyped simultaneously (Höijer et al. 2018). This complicates assessing the pathogenic character, as only the polyglutamine (CAG) repeat is known to expand and cause disease. The flanking polyproline (CCG) fragment at this locus is stable but could confuse the genotyping tools while influencing the polyglutamine pathogenicity (Urbanek et al. 2020). For these reasons, pathSTR will display a warning when the pathogenic length is added to the length plots. The pathogenic length can also be evaluated in the “sequence” mode of the repeat composition section.

Sequence composition is another important determinant of pathogenicity. As reported before, a high motif heterogeneity can be observed for the pentamer repeats (Rajan-Babu et al. 2024). A clear example includes the intronic pentamer repeats in the *YEATS2* gene (Supplemental Fig. S7), one of the causes of familial adult myoclonic epilepsy (FAME4). Only expanded alleles with an ATTTC motif are pathogenic for this locus. In this instance, evaluating patients on the overall repeat length alone is insufficient as expansions of ATTTC are pathogenic, but expansions of the reference sequence ATTTT are seen in healthy individuals (Depienne and Mandel 2021). Flanking imperfect repeat motifs can also be identified in pathSTR, depending on the repeat coordinates and the surrounding sequence. In contrast, other repeats, such as those in the *DM1*, *GLS*, and *DAB1* loci, are highly uniform in composition. Given the variable sequencing coverage in these cohorts, low-level sequencing inaccuracies can be identified in the sequence composition, whereas known motif interruptions such as those in the *FMR1* repeat can be readily identified (Fig. 3).

Similarly to the “sequence” repeat composition visualization or aSTRonaut plots, the TRGT genotyper for tandem repeats from PacBio data includes a subcommand for the visualization per individual for motif sequences and interruptions, but without the ability to dynamically interact with the visualization. Recent publications include a similar visualization as the “collapsed” repeat composition heatmap (Dolzhenko et al. 2024; Gustafson et al. 2024), but the dynamic options to customize and filter the result in pathSTR are highly valuable, as well as the large underlying data set. We are likely still lacking the full picture of the sequence compositions of these tandem repeats as long-read methods have only recently started probing the composition of expanded alleles. Therefore, unbiased genotyping regarding the expected motifs in STRdust and LongTR is highly important. A more complete view of the sequence composition in expanded repeats of reference individuals and clinical cases will improve our understanding of what makes repeats pathogenic, eventually leading to better diagnostics.

The current data set does not provide information on the DNA methylation status, which can be determined from nanopore sequencing as native DNA is sequenced without amplification (Giesselmann et al. 2019). This would be a very relevant layer of information to incorporate in a later update, as especially long CG-rich repeats are known to be methylated and lead to epigenetic silencing in *cis* (Depienne and Mandel 2021). This resource will continuously expand when new population sequencing efforts are made available, novel compatible tandem repeat genotypers are released, or additional tandem repeats are identified as relevant for human diseases.

## Methods

### Quality control

We used cramino for quality control and to determine library metrics such as library N50, yield, and normalized coverage per chromosome (De Coster and Rademakers 2023). Samples with an estimated coverage of <10× (32 Gb) were removed as a heuristic to remove the lowest informative samples in the data set (N = 129) (Supplemental Fig. S8). We additionally removed a sample with an unexpected normalized coverage on the sex chromosomes (Supplemental Fig. S9).

### Genotyping medically relevant tandem repeats

Tandem repeats for pathSTR from public resources of The 1000 Genomes Project were genotyped with STRdust (v0.8; see below) and LongTR (v1.0; adapted to access remote alignment files) (Ziaei Jam et al. 2024). The user can specify which genotyping tool (STRdust or LongTR) and genome build (GRCh38 or T2T-CHM13v2.0) to use for visualization in the web tool. The repeats with a role in human diseases selected for genotyping were taken from STRchive (Hiatt et al. 2024), using the GRCh38 and T2T coordinates for genotyping, the motif length for *k*-mer composition plots, and the provided cutoff for repeats to be considered pathogenic. STRdust implements a ‐‐pathogenic option to download tandem repeat coordinates from STRchive for ease of genotyping these medically relevant tandem repeats. Genotyping The 1000 Genomes Project samples is organized using the Snakemake workflow manager (Köster and Rahmann 2012).

### STRdust repeat genotyping

STRdust is implemented in Rust and uses the rust-htslib and rust-bio crates (Köster 2016). STRdust is implemented in such a way that alignment files (in CRAM or BAM format) do not have to be available locally but can instead be queried from a remote location (using FTP, HTTPS, or s3), which is relevant in the context of this application. At first, STRdust will collect reads that overlap with the coordinates of the repeat locus, optionally leveraging prephased alignments based on, for example, LongShot (Edge and Bansal 2019) or WhatsHap (Martin et al. 2016) or similar tools that add the HP sam tag to alignments. These reads are subsequently aligned to an artificial reference sequence of which the repeat sequence has been excised, using the rust bindings to minimap2 (Li 2021; https://github.com/jguhlin/minimap2-rs). The obtained insertions relative to the artificial reference are assumed to completely represent the repeat allele, after which a consensus of the repeat allele is generated using a partial overlap alignment (SPOA) (Vaser et al. 2017), as implemented in rust-bio. In the absence of phasing information, STRdust will perform pairwise alignment of the insertions and hierarchical clustering to identify the reads that make up the two alleles, to assign a heterozygous or homozygous genotype. Leaves connected to the highest node in the tree representing <10% of the reads while having a dissimilarity of more than five with the parent node are excluded, as those are typically low-quality reads or sequences that are not repetitive. Reads with a repeat length of twice the median of the repeat lengths of the larger allele are reported as outliers. STRdust is compatible with haploid sex chromosomes and will not attempt to split reads into two haplotypes for haploid sex chromosomes in individuals with one copy of the X Chromosome. Commandline arguments are parsed with clap (https://github.com/clap-rs/clap), and parallelization is achieved using rayon (https://github.com/rayon-rs/rayon). Binaries for STRdust are available at GitHub (https://github.com/wdecoster/STRdust), and the source code is available under the MIT license. A very early implementation of STRdust was conceived as part of the Third Annual Baylor College of Medicine & DNANexus Structural Variation hackathon (Walker et al. 2022).

### Genotyping comparison

We compared STRdust (v0.8.1) to LongTR (v1.0) for the set of medically relevant tandem repeats for ONT data of the Genome in a Bottle sample (HG002, giab_2023.05; obtained from https://registry.opendata.aws/ont-open-data/). Commands used for genotyping are provided in the Supplemental Methods. We used a scatter plot matrix to evaluate the correlation of the obtained repeat allele lengths with the tandem repeat benchmark (English et al. 2024) and calculated the Pearson correlation coefficient. Additionally, for all samples in the cohort, we compared the genotypes obtained for STRdust and LongTR, and for STRdust, we compared the genotypes between alignments to the GRCh38 reference against alignments to the T2T assembly.

### PathSTR web app

The pathSTR web app is written in Python and built upon dash (https://dash.plotly.com/) and additionally uses cyvcf2 to parse VCF files (Pedersen and Quinlan 2017), pandas to manipulate data frames (McKinney 2011), rustworkx to generate the graph for run-length encoding (Treinish et al. 2022), and modules from the Python standard library (https://www.usenix.org/conference/2007-usenix-annual-technical-conference/presentation/python-programming-language). The parsed data are saved into an hdf5 container for easier access and quick start-up times (https://github.com/HDFGroup/hdf5). For every repeat locus and individual, an IGV visualization is provided using igv.js (Robinson et al. 2023) as made available through dash-bio (Hossain 2019).

A run-length encoding is also provided for each repeat allele, for example, (GGC)_15_ or CCA(GCA)_11_, as a means to assess the repeat sequence. The repeat composition is visualized by counting *k*-mers/motifs (with the motif length k determined by the tandem repeat finder annotation of the locus) in the repeat sequence according to the forward strand of the reference genome, splitting sequences based on the known repeat motif length but unbiased to which motifs can be found. Each *k*-mer is rotated (GCA-CAG-AGC) and represented by either the known unit (as defined by STRchive) or the lexicographically first motif of the rotations. The “sequence” visualization will assign a color to the 10 most frequently observed motifs, with the remainder left in gray for inspection (Supplemental Fig. S10). Motifs seen too rarely are discarded from the “raw” and “collapsed” heatmaps, as we assume those are sequencing noise, and unfiltered visualization of too many motifs is untractable. Specifically, in the “raw” mode, motifs seen for <1% of the sequence in <2% of the alleles are discarded, except if an allele has at least 10% of their sequence composed of this rare motif. The “collapsed” mode performs a more aggressive filtering to get larger groups. Instead, it will remove motifs observed for <5% of the sequence in 10% of the individuals, except if five alleles contain at least 10% of this rare motif. This data-driven approach enables the visualization of motif changes and interruptions while reducing random sequencing noise.

The pathSTR web app is hosted in-house and deployed using Nginx and Gunicorn. The code of the pathSTR app is open source and available under the MIT license at GitHub (github.com/wdecoster/pathSTR). Scripts and jupyter notebooks for comparing genotypers and alignment to GRCh38 and T2T are available in the repository, as well as instructions on genotyping, constructing the pathSTR database, and launching the web app.

### Targeted nanopore sequencing

According to the manufacturer's protocol, 5 µg of genomic DNA was extracted from whole blood for Cas9-targeted sequencing using the SQK-CS9109 kit (ONT). Coordinates and sequences targeted with Cas9 are available in Supplemental Table 1. Nanopore sequencing was performed on a MinION R9.4.1 flowcell (ONT) at the SciLifeLab National Genomics Infrastructure (NGI) in Uppsala, Sweden. Basecalling was done with Guppy (v6.3.8, Super-accurate basecalling with base modifications 5mC context, MinKNOW, ONT), followed by alignment to the GRCh38 reference genome with minimap2 (v2.24-r1122, with parameters -H -ax map-ont).

### Ethics declaration

The Swedish ethical review authority (2019-04746) approved the study concerning the DM1 patient, and written informed consent was obtained from each participating individual or their respective legal guardians.

## Data access

The data generated in this project can be accessed at https://pathstr.bioinf.be, where the data can be queried, visualized, and downloaded as a tab-separated file or individual VCF files generated by STRdust or LongTR for alignments against the GRCh38 reference or the T2T assembly. The alignments used in this work are publicly available at https://ftp.1000genomes.ebi.ac.uk/vol1/ftp/data_collections/1KG_ONT_VIENNA/ and https://s3.amazonaws.com/1000g-ont/index.html. All code (pathSTR, custom scripts and notebooks) at the time of publication is archived in the Supplemental Code as a zip file of the pathSTR repository.

## Supplemental Material

Supplement 1

Supplement 2
