# Supplementary material for: Visualization and analysis of medically relevant tandem repeats in nanopore sequencing of control cohorts with pathSTR
Source: Genome Res. 2024 Nov;34(11):2074–80. doi: 10.1101/gr.279265.124 (PMC11610575; doi:10.1101/gr.279265.124)
Supplement: Supplement 1 [file Supplemental_Material.pdf]

Supplementary Data

Supplementary Tables

Supplementary Table 1: Cas9 target sites for the targeted nanopore sequencing of the *DMPK* repeat locus.

Target sequence (incl. PAM)	Genomic location	gRNA ID	Strand
GGAGAGCGGTACCACTTGTGGGG	chr19:45,766,588-45,766,610	DMPK_FWD1	+
AGGTTCACGTTTCACAACAAAGG	chr19:45,767,591-45,767,613	DMPK_FWD2	+
AAGTTTCGGTGGATCATTCCAGG	chr19:45,767,247-45,767,269	DMPK_FWD3	+
CGGACAACCAGAACTTCGCCAGG	chr19:45,771,770-45,771,792	DMPK_REV1	-
CGTGTATAGACACCTGGAGGAGG	chr19:45,771,480-45,771,502	DMPK_REV2	-
GACGAGGTTACTTCAGACATGGG	chr19:45,772,489-45,772,511	DMPK_REV3	-

Supplementary Methods

Commands used for benchmarking STRdust and LongTR on HG002 data:

LongTR --bams {input.cram} --bam-samps {sample_id} --bam-libs {sample_id} --fasta genome_hg38.fa --haploid-chrs chrX,chrY --regions STRchive_LongTR.bed --min-sum-qual -1e10 --tr-vcf {output.vcf}

STRdust --haploid chrX,chrY --region-file hg38.STRchive-disease-loci.TRGT.bed genome_hg38.fa {input.cram}> {output.vcf}

The script for comparison of these genotypes is available in the pathSTR repository at https://github.com/wdecoster/pathSTR/blob/main/scripts/splom_comparison.py

The command used to generate Supplementary Figure S6:
python scripts/aSTRonaut.py --somatic --repeat chrX:147912037 --motifs CGG,AGG,AGC,CGT -o aSTRonaut_somatic.html --publication HG02389.vcf.gz --size 8

Supplementary Figures

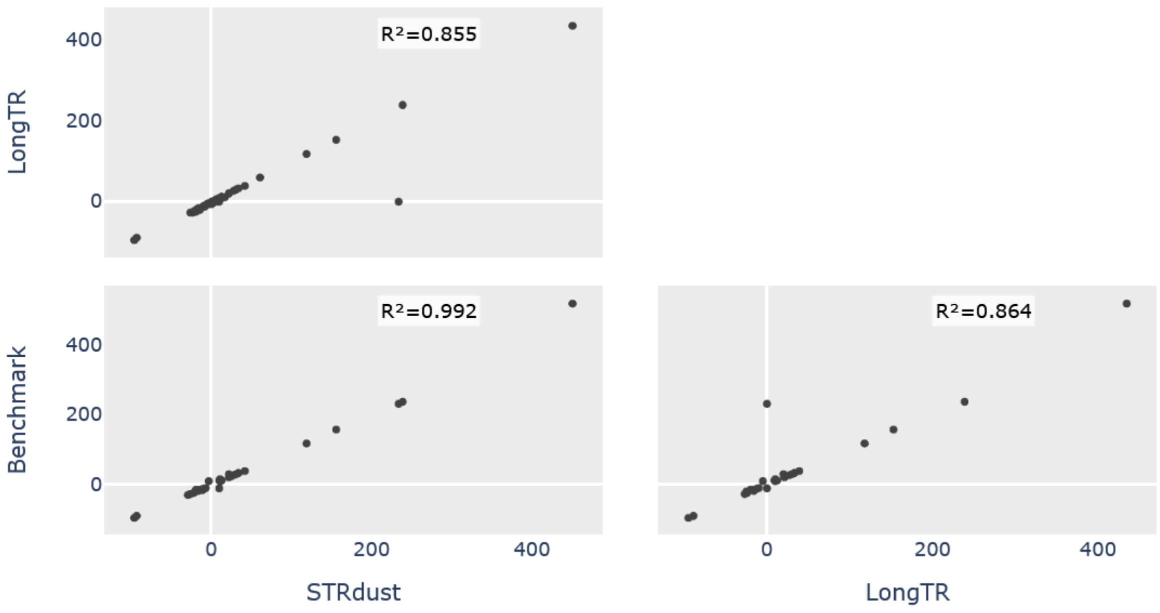

Supplementary Figure S1: correlation of medically relevant tandem repeat lengths between STRdust v0.8.1 and LongTR v1.0, compared to the HG002 benchmark lengths (English et al. 2023), with each dot the repeat allele length of a locus. The R^2 value is the Pearson correlation coefficient.

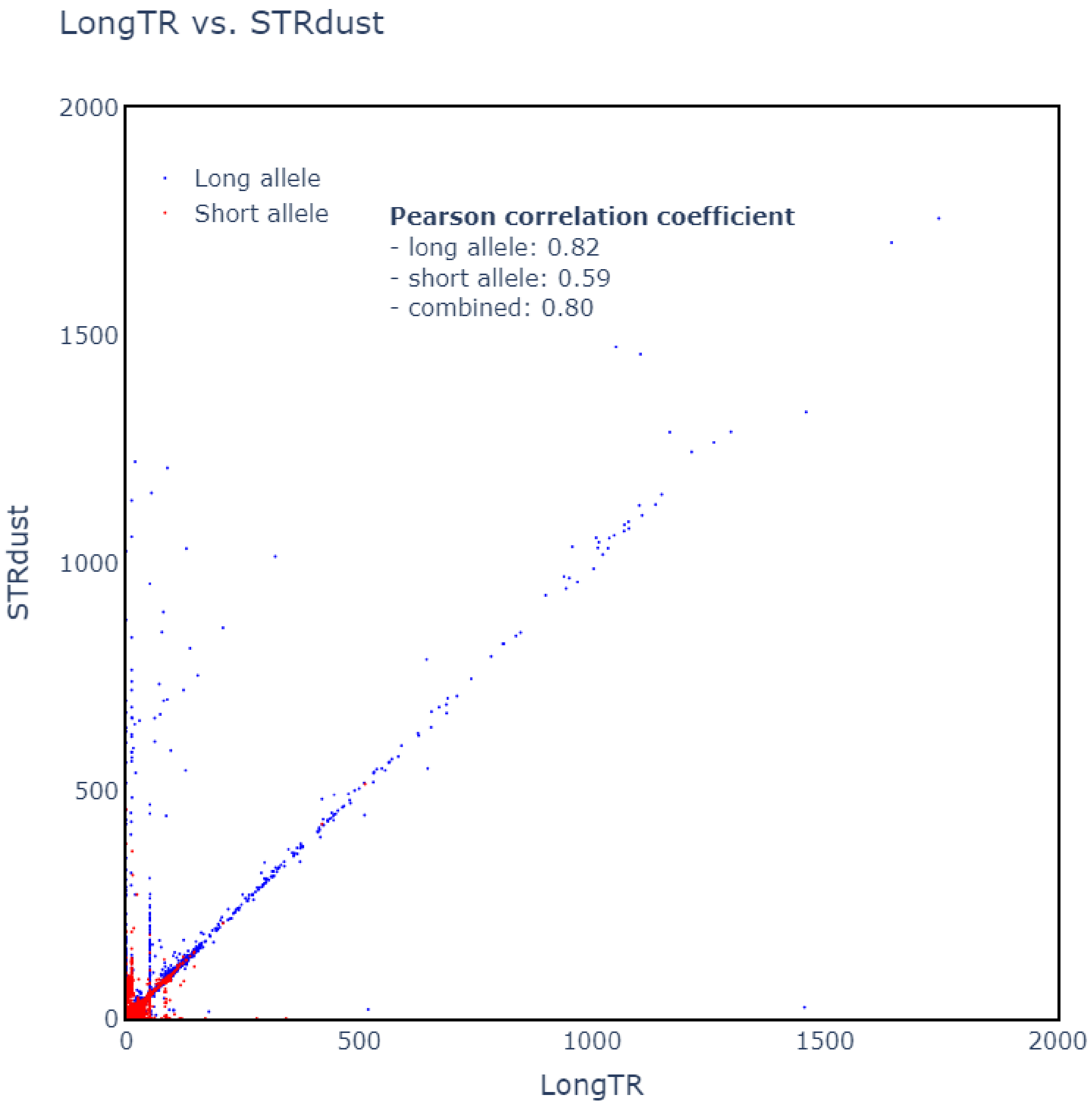

Supplementary Figure S2: correlation of medically relevant tandem repeat lengths between STRdust v0.8.1 and LongTR v1.0 across the entire cohort, with each dot the repeat allele length of a locus.

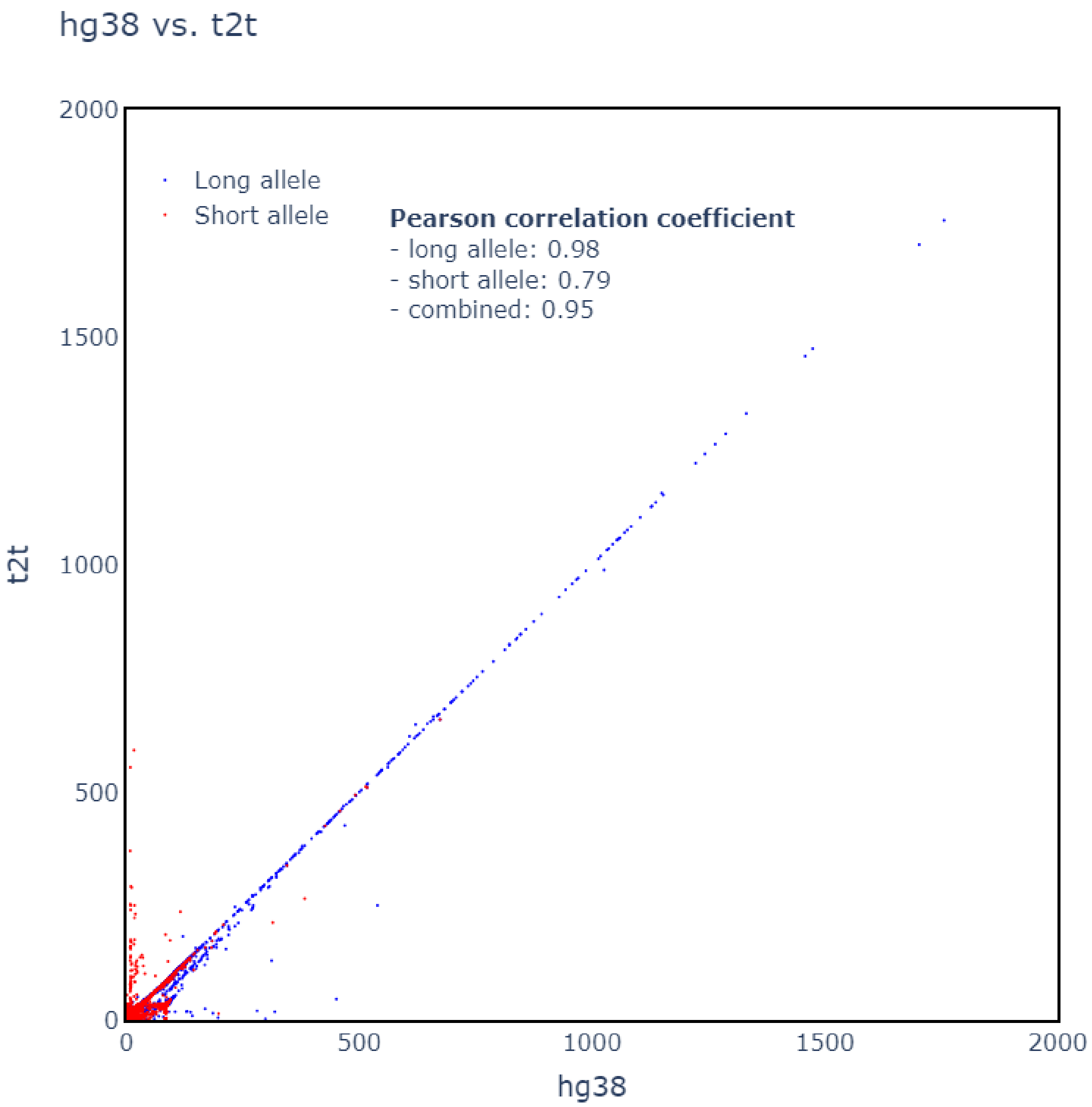

Supplementary Figure S3: correlation of medically relevant tandem repeat lengths between alignments against the hg38 reference and the T2T assembly for STRdust v0.8.1 across the entire cohort, with each dot the repeat allele length of a locus.

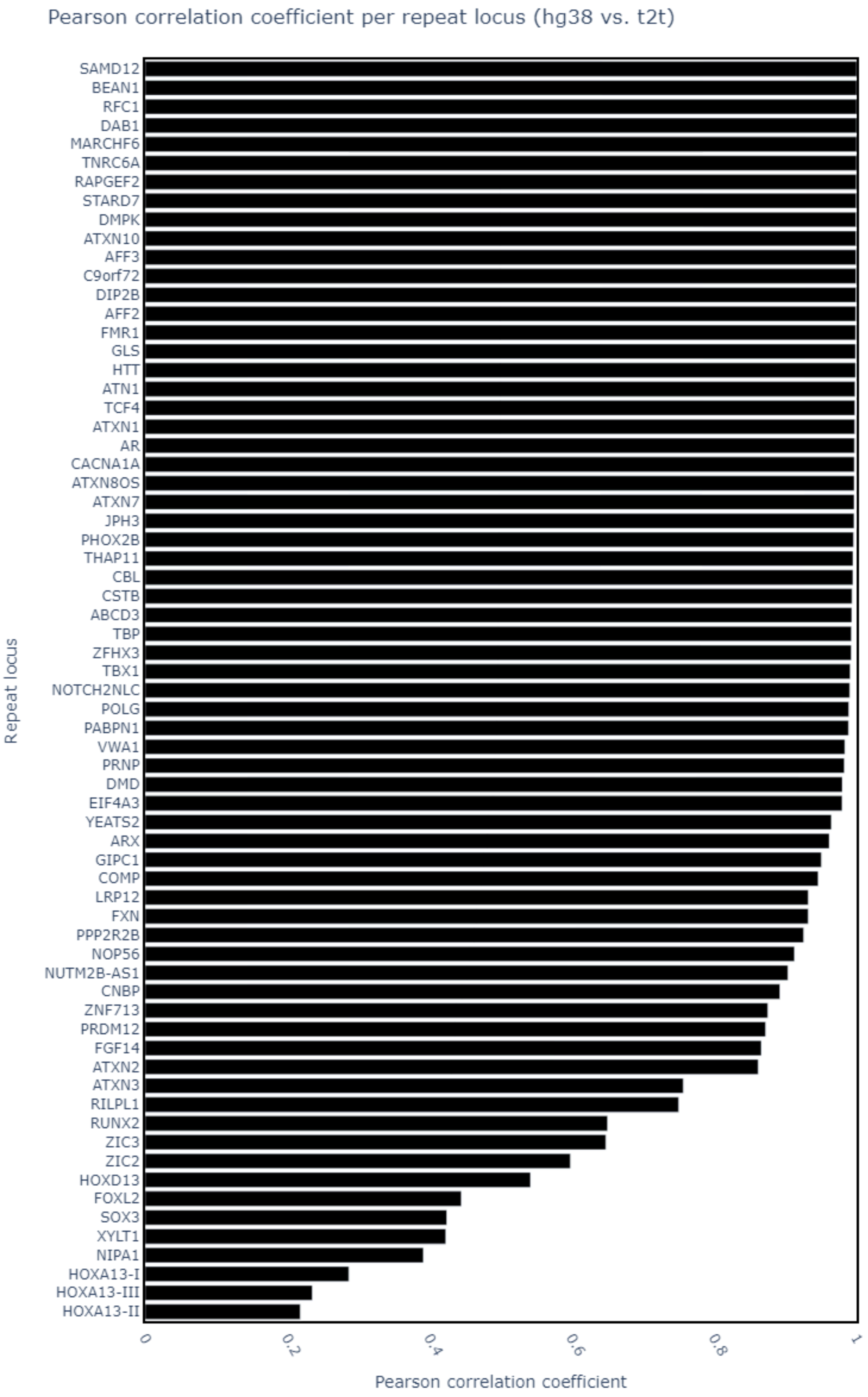

Supplementary Figure S4: correlation coefficient per gene of medically relevant tandem repeat lengths between alignments against hg38 and T2T for STRdust v0.8.1 across the entire cohort.

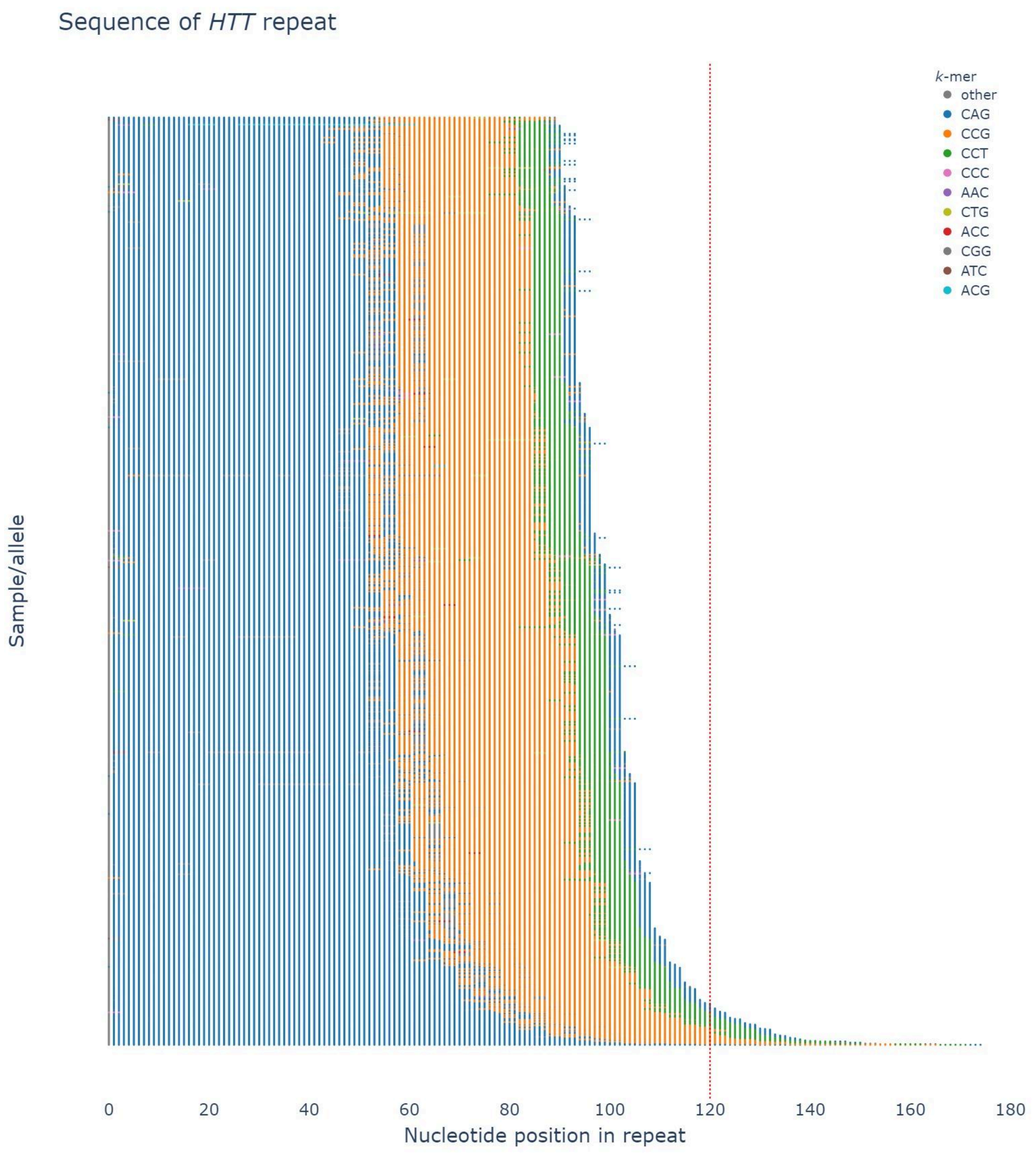

Supplementary Figure S5: repeat sequence visualization, showing the composition of the *HTT* repeat (minimum allele length cutoff for visualization of 30 units), with CAG motifs in blue and the pathogenic cutoff as a vertical red line. This indicates one individual, HG02275, with a pathogenic expansion.

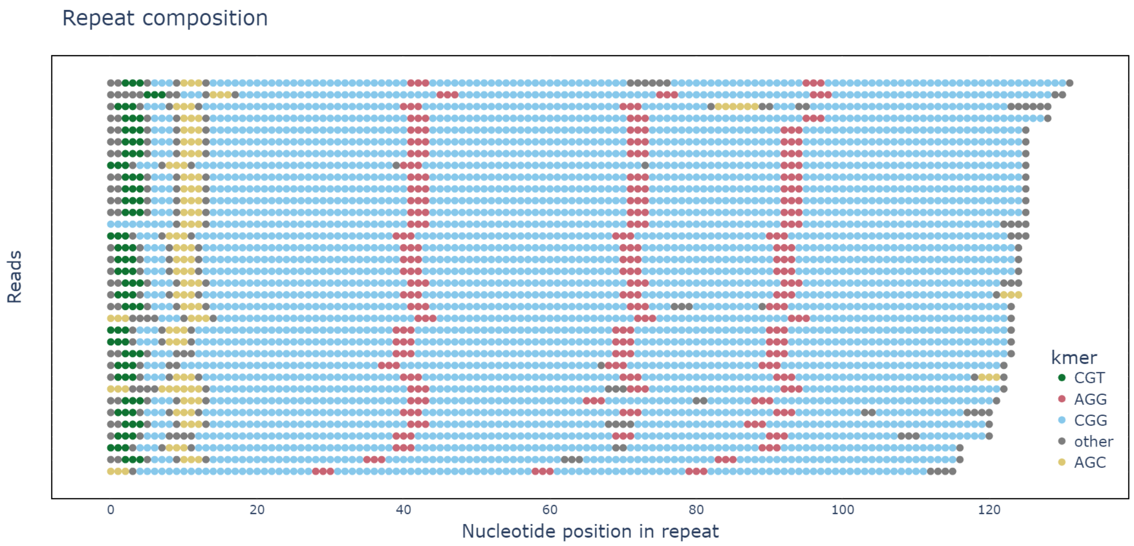

Supplementary Figure S6: *sequence* plot using the aSTRonaut companion script for somatic differences in the *FMR1* repeat of HG02389, showing the expansion sequence for every individual read.

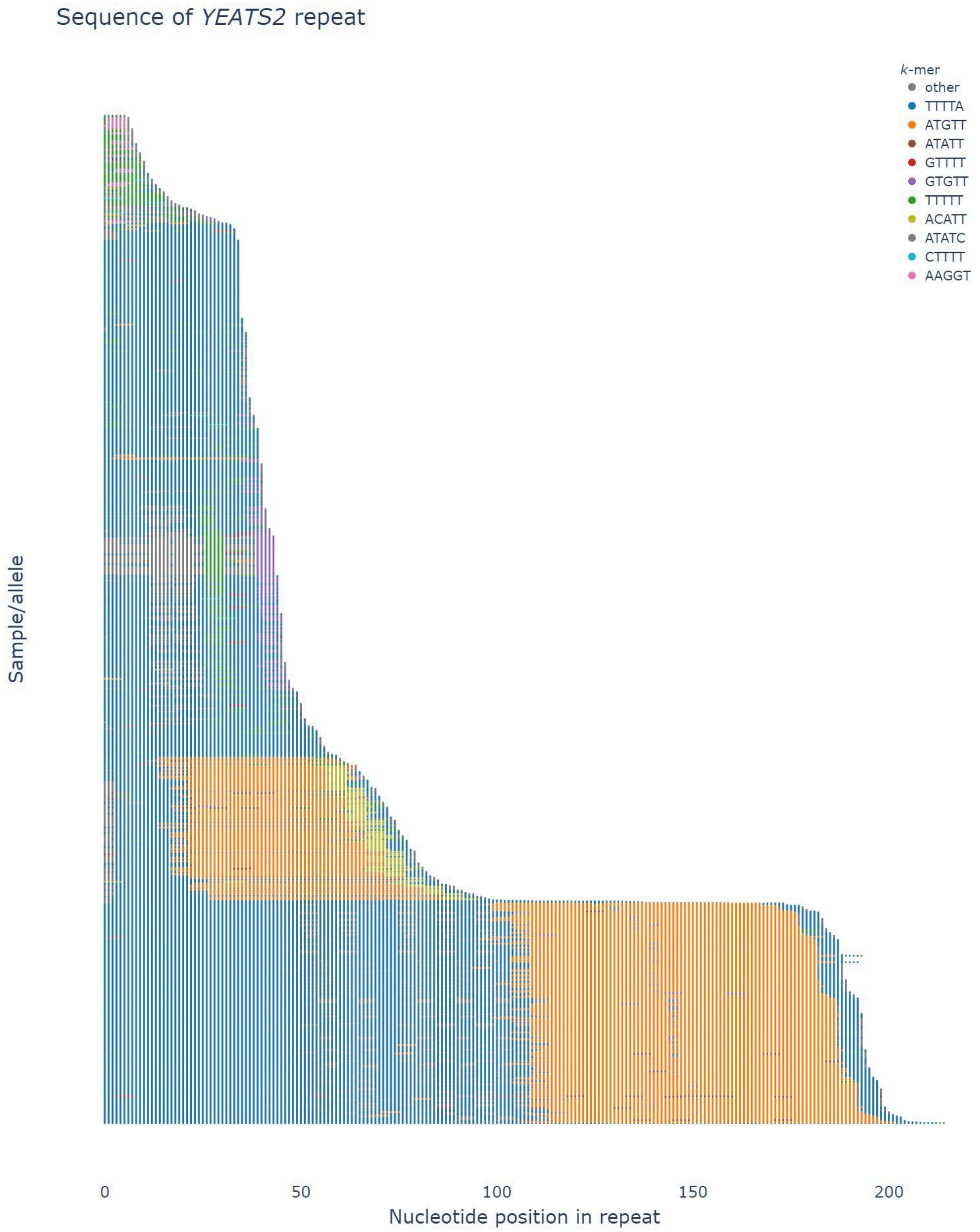

Supplementary Figure S7: *sequence* plot of the *YEATS2* repeat, showing colors for the most frequently seen motifs and grey for everything else, sorted by length.

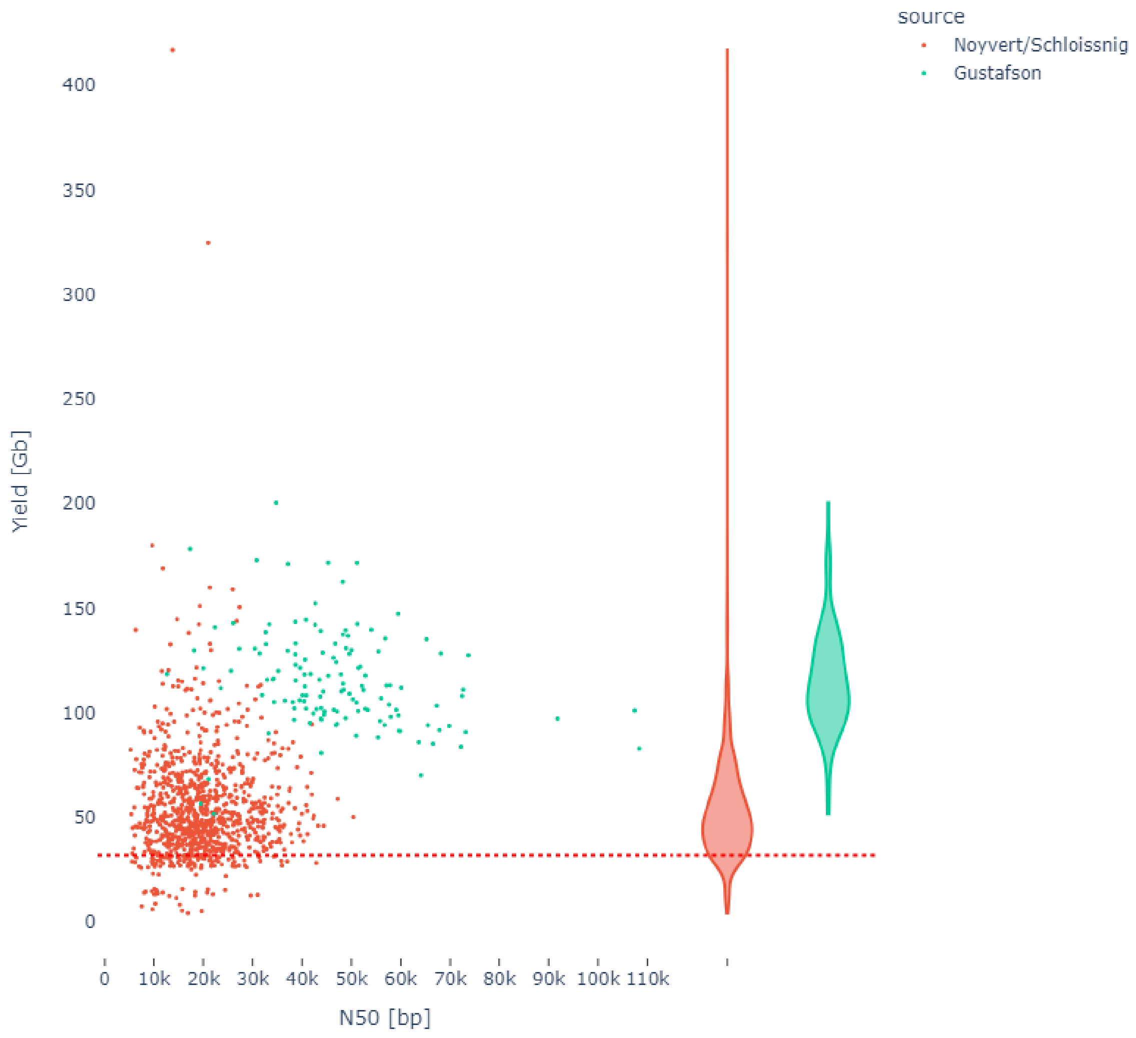

Supplementary Figure S8: scatter plot of sequencing yield and N50 library length, with a marginal violin plot for yield and a horizontal line for the minimum yield cutoff (32Gb), corresponding to an estimated coverage of 10x.

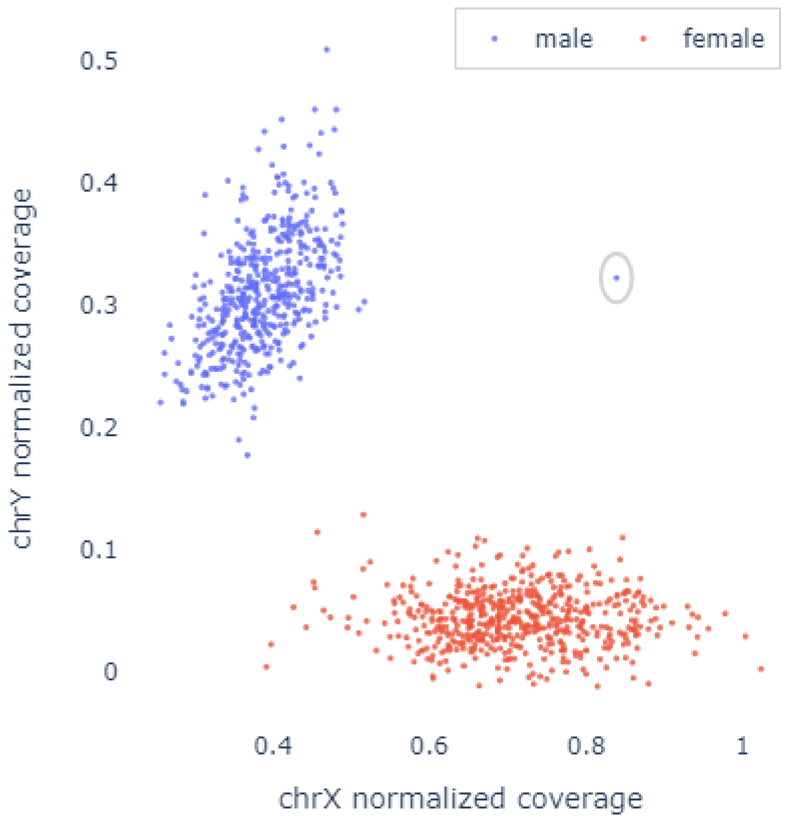

Supplementary Figure S9: visualization of sex chromosome dosage, with a circle around one suspected carrier of Klinefelter syndrome (XXY, HG02372). Points are colored based on the sex as provided in the sample info file downloaded from https://www.internationalgenome.org/data-portal/sample

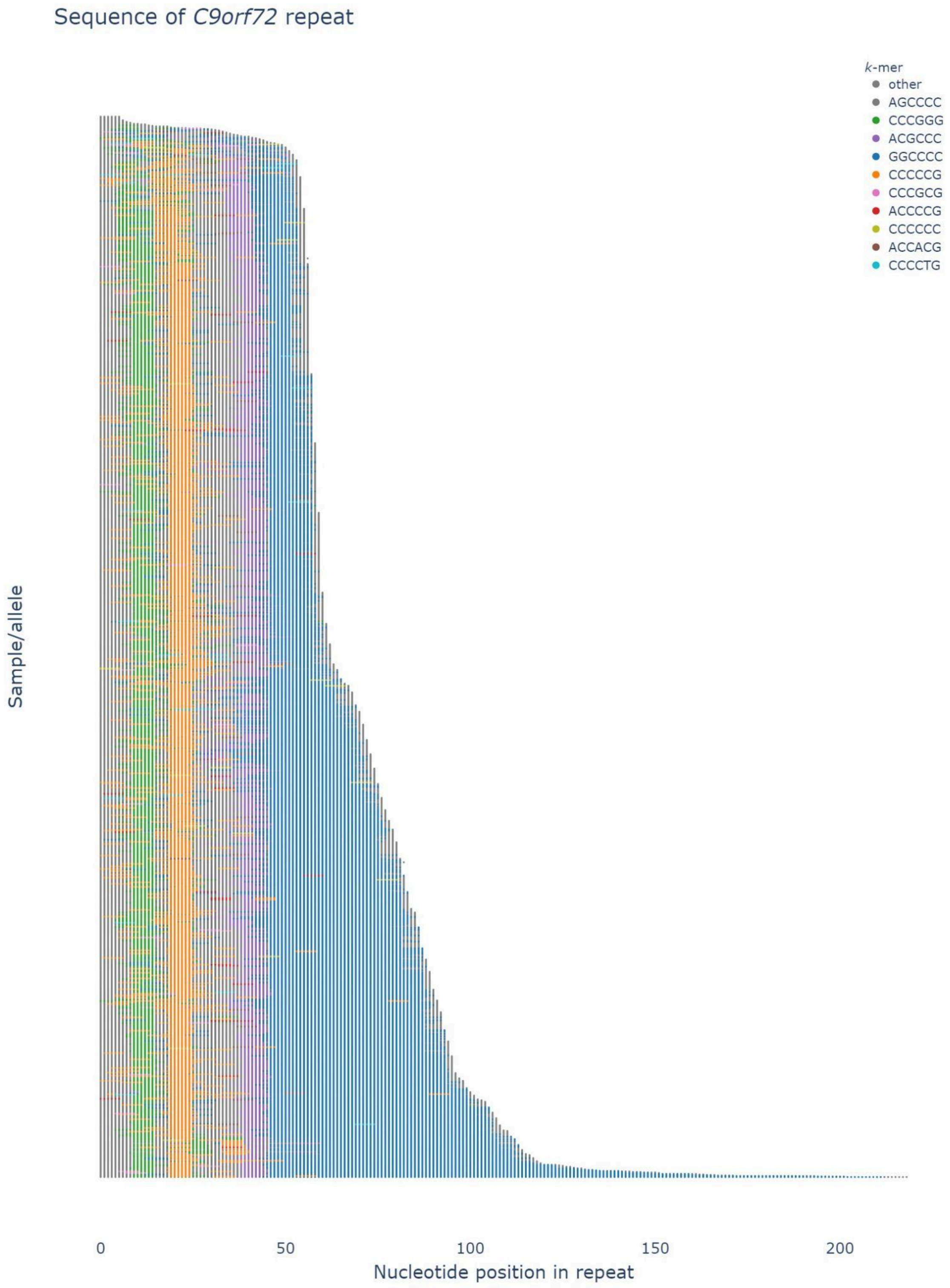

Supplementary Figure S10: *sequence* plot of the *C9orf72* repeat, showing colors for the most frequently seen motifs and grey for everything else, sorted by length.
