# Supplementary figures and images for: Visualization and analysis of medically relevant tandem repeats in nanopore sequencing of control cohorts with pathSTR
Source: Genome Res. 2024 Nov;34(11):2074–80. doi: 10.1101/gr.279265.124 (PMC11610575; doi:10.1101/gr.279265.124)

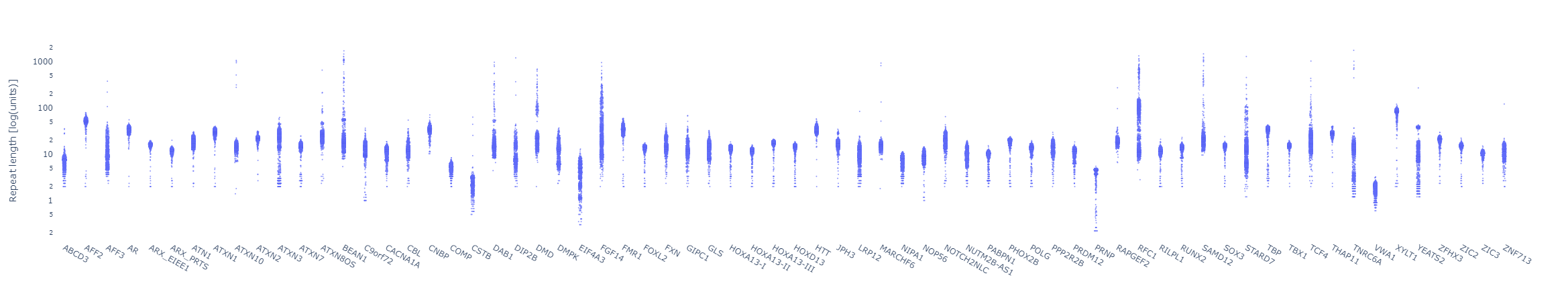

Supplement: Supplement 2 [file Supplemental_Code.zip › pathSTR-main/pathSTR-main/assets/overview-strip.png]
